# Addressing gender inequalities in music theory education: a gender-responsive flipped classroom framework

**DOI:** 10.3389/fpsyg.2026.1833228

**Published:** 2026-05-07

**Authors:** Qisen Zhu, Jinglong Li

**Affiliations:** 1 Faculty of Education, University Kebangsaan Malaysia, Selangor, Malaysia; 2 Department of Industrial Design, Faculty of Design and Architecture, University Putra Malaysia, Serdang, Malaysia

**Keywords:** flipped classroom, gender equity, inclusion, music education, self-directed learning

## Abstract

While music education plays a vital role in shaping learners' identities and participation, persistent gender inequalities continue to influence engagement, particularly in music theory education, where abstract content and traditionally lecture-dominated pedagogies may reinforce unequal participation. Although flipped classroom (FC) and self-directed learning (SDL) are widely recognized for enhancing engagement and learner autonomy, their application has largely remained generic and insufficiently responsive to the discipline-specific cognitive demands of music theory and to gendered participation patterns. To address this gap, this study develops a gender-responsive FC–SDL framework tailored to undergraduate music theory education. Drawing on a structured literature synthesis of 46 studies published between 2015 and 2025 and employing iterative thematic analysis, the study identifies key instructional, learner-related, and contextual mechanisms shaping engagement and participation. The findings indicate that learning in music theory is grounded in discipline-specific tasks—including harmonic analysis, counterpoint construction, and aural training—which require the integration of symbolic reasoning, auditory perception, and rule-based processing. At the same time, gendered differences in confidence, participation, and self-regulation function as mediating mechanisms that influence how learners engage with these tasks and may reproduce inequalities if not explicitly addressed in instructional design. Building on these insights, the proposed framework integrates task-specific learning processes with gender-responsive instructional strategies, advancing a mechanism-based and discipline-sensitive model. This study offers a theoretically grounded and practically applicable framework for promoting equitable participation and effective learning in music theory education.

## 1 Introduction

Despite growing emphasis on learner diversity and inclusive education, gender-based disparities remain deeply embedded within music education, shaping students' participation, confidence, and access to learning opportunities. These disparities extend beyond observable differences in engagement to reflect socially constructed norms that influence instrument selection, musical identity, and classroom interaction patterns (Green, 1997; Koza, 2009; Abeles, 2009). Furthermore, such inequalities are inherently intersectional, interacting with socio-economic background, cultural context, and access to educational resources, thereby producing differentiated learning experiences across student groups (Crenshaw, 2013). In music theory education, these issues are particularly pronounced due to the abstract and cognitively demanding nature of the subject, which may amplify disparities in confidence, prior knowledge, and participation. These disparities are increasingly understood within gender theory as socially constructed and interactionally reproduced, rather than as fixed individual differences.

Within this context, flipped classroom (FC) and self-directed learning (SDL) have been widely promoted as learner-centered approaches capable of enhancing flexibility, engagement, and autonomy in higher education (Lo and Hew, 2017; Ozer and Yukselir, 2023). By redistributing instructional time and emphasizing active learning, these pedagogies are often assumed to democratize access to knowledge and support diverse learners. However, such assumptions remain insufficiently examined. The effectiveness of FC and SDL relies heavily on learners' self-regulation, motivation, and access to resources—capacities that are unevenly distributed and may be shaped by gendered socialization processes and structural constraints (Yaghoubi et al., 2025). Consequently, rather than inherently reducing inequality, these approaches may inadvertently reproduce or even intensify existing disparities when issues of diversity and inclusion are not explicitly addressed.

Although prior studies have demonstrated the pedagogical benefits of FC and SDL, research has largely focused on general learning outcomes such as engagement and academic performance, with limited attention to how these approaches interact with gendered participation patterns and intersectional inequalities in discipline-specific contexts (McKenney and Reeves, 2025; Spangler, 2025). In particular, little is known about the mechanisms through which instructional design and learner autonomy jointly shape equitable participation in music theory education, where diverse learner entry points and abstract conceptual demands present unique challenges. The absence of a theoretically integrated and gender-responsive framework therefore represents a critical gap in both music education and technology-enhanced learning research.

In response to this gap, this study aims to develop a gender-responsive flipped classroom framework that integrates self-directed learning to promote equitable participation and inclusive learning experiences in undergraduate music theory education. Unlike prior studies that treat flipped classroom and self-directed learning as generic pedagogical approaches, this study advances the literature by developing a mechanism-based framework that explicates how instructional design activates cognitive–behavioral processes leading to learning outcomes. The framework further strengthens domain specificity by embedding music theory–specific tasks within the pedagogical structure, ensuring alignment with the cognitive and procedural demands of the discipline. Moreover, gender is reconceptualized not as a demographic attribute but as a set of gender-responsive instructional mechanisms that shape participation, interaction, and feedback processes. Through this integration, the study offers a more theoretically grounded and context-sensitive model for understanding and supporting equitable learning in music theory education. By situating instructional design within a gender-aware and intersectional perspective, this study seeks to advance theoretical understanding of how pedagogical structures interact with learner diversity while providing practical guidance for reducing gender-based disparities in music education contexts.

## 2 Methodology

**Figure 1:**
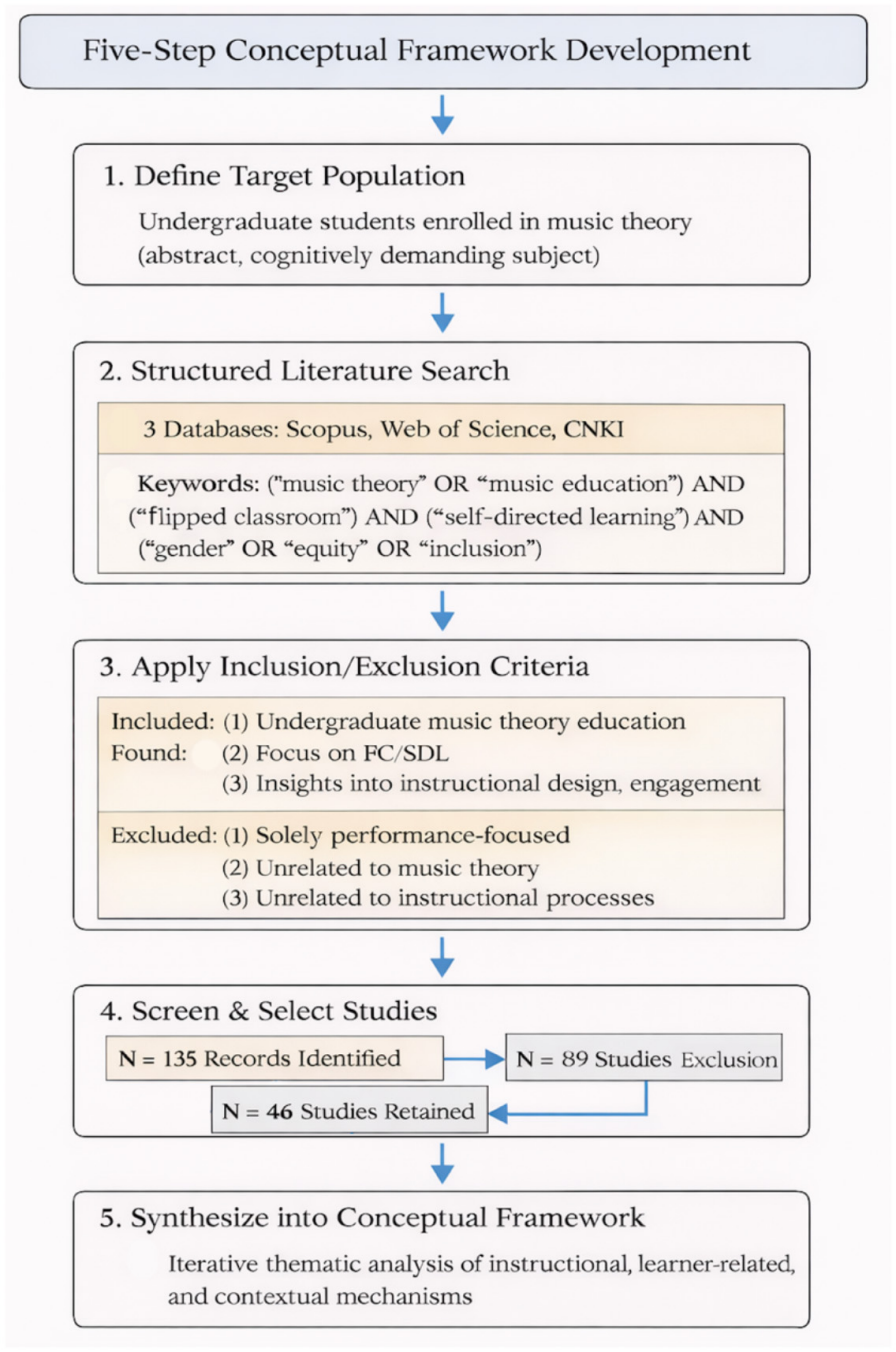
structured literature synthesis and framework development process for gender responsive FC-SDL model.

This study adopts a conceptual framework development design informed by a structured literature synthesis approach, with the aim of developing a coherent and mechanism-based framework for music theory education (Bharti et al., 2015). Positioned as a theory-building study, it focuses on the identification, integration, and conceptual organization of key constructs, rather than conducting a full systematic review or empirical investigation. In contrast to systematic review methodologies that emphasize exhaustive coverage and formal quality appraisal, this study prioritizes the purposeful selection and conceptual integration of relevant literature to support framework construction. To ensure methodological rigor, transparency, and analytical coherence, a structured five-step procedure was employed (see Figure 1), establishing a logically connected and closed methodological pathway that links literature identification, thematic abstraction, and framework synthesis within a unified research design.

The first step involved defining the target population as undergraduate students enrolled in music theory courses in higher education. This step provides the contextual foundation for the study by situating the framework within a discipline characterized by abstract conceptual structures and complex cognitive demands, including the integration of symbolic reasoning, auditory processing, and rule-based understanding. By clearly specifying the learner context, the framework development process is anchored in the pedagogical and cognitive characteristics unique to music theory education. This context-sensitive positioning further supports the study's emphasis on conceptual integration, ensuring that the selected literature and subsequent analytical processes are aligned with the objective of constructing a theoretically coherent and domain-relevant framework.

In the second step, a structured literature synthesis was conducted across three major academic databases: Scopus, Web of Science, and CNKI. The search strategy was guided by Ibrahim's research structuring framework and implemented using Boolean keyword combinations (Ibrahim, 2008), including: (“music theory” OR “harmonic analysis” OR “counterpoint” OR “aural skills” OR “ear training”) AND (“flipped classroom”) AND (“self-directed learning”) AND (“gender” OR “equity” OR “inclusion”). Studies published between 2015 and 2025 were considered. This stage ensures systematic identification of relevant literature while maintaining a focused scope aligned with the study's conceptual objectives.

To enhance transparency and reproducibility, explicit inclusion and exclusion criteria were applied during the screening process. Studies were included if they (1) addressed higher education contexts, (2) examined flipped classroom and/or self-directed learning approaches, and (3) provided empirical or conceptual insights into instructional design, learner engagement, or participation. Studies were excluded if they (1) focused solely on performance or instrumental training, (2) were unrelated to music theory or higher education, or (3) lacked relevance to instructional processes. Following this process, a total of 46 studies were retained for in-depth analysis. This step ensures that the selected literature is both relevant and conceptually aligned with the research objectives.

In the third step, an iterative thematic analysis was conducted to systematically extract and organize key constructs. This process involved open coding of the selected studies, followed by category refinement and abstraction into higher-order themes. The resulting constructs were organized into three interrelated dimensions: instructional, learner-related, and contextual. This analytical stage enables the transition from descriptive literature review to conceptual structuring.

In the fourth step, the relationships among the identified constructs were examined to uncover underlying mechanisms shaping engagement and participation. This stage adopts a relational and interpretive approach, integrating a gender-responsive and intersectional perspective to explain how individual, instructional, and contextual factors interact within music theory learning environments. By moving beyond isolated variables, this step provides a mechanism-based understanding of learning processes.

In the 50th step, the identified constructs and their interrelationships were synthesized into a coherent conceptual framework, forming the proposed FC–SDL model. This final stage integrates insights from the literature synthesis and thematic analysis into a structured and theoretically grounded model, ensuring alignment between empirical evidence, analytical procedures, and framework design.

Taken together, these 5 steps establish a clear methodological progression from literature identification to conceptual synthesis, forming a closed and logically coherent analytical pathway that enhances the transparency, rigor, and credibility of the study.

## 3 Results

### 3.1 The current situation and needs of undergraduate students in music theory programs in higher education

The findings of this study indicate that music theory education in higher education remains predominantly shaped by teacher-centered instructional approaches, characterized by limited pedagogical diversity and insufficient innovation. Instructional practices are largely confined to lecture-based knowledge transmission, where teachers reproduce textbook content with minimal interaction, resulting in passive learning environments and constrained opportunities for critical engagement and creative expression (Al-Zahrani, 2015). Such approaches are particularly problematic in music theory education, an inherently artistic and conceptual domain that requires not only cognitive understanding but also experiential engagement and creative interpretation (Gaunt et al., 2021). Consequently, students' motivation and sustained engagement are often weakened, leading to reduced interest and limited depth of learning (Ferrer et al., 2022).

The analysis further reveals that the lack of diversified instructional strategies—including interactive discussion, multimedia integration, and creative practice—significantly restricts students' engagement and learning effectiveness (Sabri et al., 2024; Yang, 2023). Students commonly perceive music theory as abstract and detached from practical musical application, which contributes to superficial understanding and low learning investment (Huovinen, 2021). This perception is reinforced by instructional approaches that emphasize memorization over conceptual understanding, thereby limiting students' ability to transfer knowledge to higher-order tasks such as analysis, evaluation, and creative production (Newman and Gentile, 2020). Moreover, the abstract and dynamic nature of music theory concepts presents additional cognitive challenges, particularly when instructional representations remain static and insufficiently explanatory (Yarden and Yarden, 2010; Kim and Steiner, 2016).

Importantly, the findings suggest that these challenges are unevenly distributed across learners and are closely associated with variations in confidence, prior knowledge, and self-regulatory capacity. Students with limited prior exposure to music theory or lower levels of self-efficacy are more likely to disengage and adopt passive learning strategies. From a gender-responsive perspective, these disparities are not merely individual but socially mediated. The analysis indicates that gendered patterns of participation and confidence may influence how learners engage with music theory, particularly in collaborative and expressive learning contexts. Learners' willingness to contribute, take risks, and express creative ideas may be shaped by socially constructed expectations and prior educational experiences. Consistent with prior research, gender-related perceptions in music learning can influence participation behaviors and confidence levels, thereby contributing to uneven engagement across learning environments (Hendricks et al., 2016). As a result, traditional pedagogical approaches may inadvertently reproduce these disparities by privileging students who are more confident or academically prepared.

The findings also identify a persistent disconnect between theoretical instruction and practical application as a central barrier to meaningful learning. Students frequently encounter difficulties in applying abstract theoretical concepts to authentic musical contexts due to insufficient integration of theory and practice. This gap is particularly pronounced for learners who require multimodal, interactive, and scaffolded learning environments to support conceptual understanding. Without such support, disparities in comprehension and participation are likely to widen, further reinforcing unequal learning outcomes.

In response to these challenges, flipped classroom (FC) approaches have been introduced as a potential means of enhancing flexibility and promoting active learning. However, the findings indicate that the implementation of FC in music theory education remains constrained by both pedagogical and structural limitations. Teachers' reliance on traditional instructional paradigms and limited familiarity with FC pedagogy hinder effective adoption (Dogan et al., 2023). At the same time, students' readiness for self-directed learning remains uneven, as learners accustomed to passive instructional modes often struggle with independent pre-class preparation and knowledge construction (Hwang and Chen, 2023). The effectiveness of FC is further influenced by resource availability and the increased demands placed on instructors in terms of instructional design and content development (Förster et al., 2022). Similarly, while technology-enhanced and online learning environments offer flexibility, they also expose limitations in students' self-regulation and autonomous learning capabilities (Vitta and Al-Hoorie, 2023).

Taken together, these findings demonstrate that challenges in music theory education extend beyond instructional design to encompass broader issues of learner diversity, participation, and access. The persistence of teacher-centered approaches, combined with unequal distributions of confidence and self-directed learning readiness, contributes to differentiated learning experiences and may reinforce existing disparities. These results underscore the need for an integrated pedagogical approach that not only enhances learning effectiveness but also explicitly addresses issues of gender, participation, and inclusion. Such insights provide a critical empirical foundation for the development of a gender-responsive flipped classroom framework that integrates self-directed learning to promote equitable participation and inclusive learning in music theory education Table 1.

**Table 1:** Key challenges and learning needs in music theory education.

Dimension	Key findings	Underlying mechanism	Implications
Instructional design	Predominance of teacher-centered approaches with limited interaction	Lack of pedagogical diversity constrains active learning	Shift toward interactive and student-centered design
Learning engagement	Low motivation: music theory perceived as abstract and disconnected from practice	Emphasis on memorization over conceptual understanding	Integrate multimodal and experiential learning
Cognitive demands	Difficulty understanding abstract and dynamic concepts	Insufficient scaffolding and visualization support	Use technology-enhanced and scaffolded instruction
Learner differences	Variations in confidence, prior knowledge, and self-regulation	Unequal learning readiness shapes participation	Provide differentiated and inclusive support
Equity and participation	Uneven participation and engagement across learners	Gendered patterns of confidence and participation may influence learning experiences	Adopt gender-responsive and inclusive pedagogy
FC–SDL readiness	Limited readiness for flipped learning and self-directed learning	Dependence on teacher guidance and weak self-regulation	Strengthen SDL competencies and structured support

These challenges are closely linked to discipline-specific learning tasks in music theory. Students frequently encounter difficulties in harmonic analysis (e.g., identifying chord functions), counterpoint writing (e.g., applying voice-leading rules), and aural skills (e.g., solfege and dictation). These tasks require the integration of abstract reasoning, auditory processing, and rule-based construction, which are not adequately supported by traditional lecture-based approaches.

### 3.2 Factors influencing undergraduate students' learning of music theory courses

Students' engagement with music theory learning is strongly influenced by their intrinsic interest in music, which functions as a key driver of learning behaviors and outcomes (Wang and Huang, 2024; Chen, 2024). The findings show that when students demonstrate a high level of interest, they are more likely to actively engage with theoretical concepts, sustain effort in cognitively demanding tasks, and develop deeper conceptual understanding. In contrast, when such interest is absent, music theory is often perceived as abstract and monotonous, leading to reduced cognitive investment and disengagement from learning activities (Westgate and Wilson, 2018).

Closely related to motivation, self-regulatory capacities play a critical role in shaping students' ability to cope with the complexity of music theory. The analysis indicates that competencies such as time management, goal setting, and independent practice are essential for navigating abstract concepts and sustaining learning progress. Students who lack these skills are more likely to experience cognitive overload and fragmented understanding, particularly when engaging with technically demanding content (Christensen, 2018). These findings suggest that learning effectiveness in music theory is not solely determined by instructional input but is closely tied to learners' capacity for self-directed engagement.

Differences in motivation, confidence, and prior knowledge further contribute to variations in participation and learning outcomes. The findings reveal that students with lower self-efficacy or limited prior exposure to music theory are more likely to adopt passive learning strategies and withdraw from active engagement. From a gender-responsive perspective, these differences are shaped not only by individual factors but also by socially mediated learning experiences. Learners' confidence, willingness to contribute, and participation in collaborative or creative activities may be influenced by gendered expectations and prior socialization processes. Existing research indicates that gendered patterns of engagement and confidence in music learning contexts can shape students' interaction with theoretical content and classroom participation, potentially leading to uneven engagement across learner groups (Hoffman, 2012). Without targeted pedagogical support, such differences may translate into unequal learning opportunities.

Instructional design plays a mediating role in determining how students engage with music theory content. The findings show that learning environments incorporating interactive, experiential, and practice-oriented activities significantly enhance students' engagement and conceptual understanding (Sun et al., 2025). When instruction integrates composition, performance, and applied problem-solving, abstract theoretical concepts become more accessible and meaningful. In contrast, lecture-dominated approaches limit opportunities for active participation and reduce the likelihood of deep learning. Instructional strategies that provide multiple modes of representation and participation are particularly important for supporting diverse learners, as they accommodate different learning preferences and reduce barriers associated with abstract content (Awang-Hashim et al., 2019).

Assessment and feedback mechanisms further shape students' learning processes by influencing motivation, confidence, and strategic learning behaviors. The findings indicate that clearly defined assessment criteria and timely, constructive feedback enable students to monitor their progress and adjust their learning strategies. Formative feedback practices are especially effective in supporting the comprehension of complex theoretical content while sustaining engagement over time. In addition, supportive feedback can enhance learners' confidence and reduce uncertainty, which is particularly important for students who may be less confident in their abilities. From an inclusion perspective, equitable feedback practices contribute to more balanced participation by providing consistent support across different learner groups and reducing performance-related anxiety.

Taken together, these findings demonstrate that students' learning of music theory is shaped by the interaction of learner characteristics, instructional design, and assessment practices. These factors operate in an interconnected manner, influencing not only cognitive engagement but also patterns of participation and confidence. When differences in motivation, self-regulation, and prior knowledge intersect with instructional and contextual conditions, they may contribute to uneven learning experiences and reinforce existing disparities. These insights highlight the importance of developing an integrated pedagogical approach that aligns instructional strategies with learner diversity while explicitly addressing issues of gender, participation, and inclusion. Such an approach provides a critical foundation for the design of a gender-responsive flipped classroom framework that integrates self-directed learning to support equitable and effective music theory education Table 2.

**Table 2:** Key mechanisms influencing music theory learning.

Dimension	Core factors	Key mechanisms	Pedagogical implications
Motivation	Intrinsic interest	Drives sustained engagement and deeper conceptual processing; lack of interest leads to disengagement	Design meaningful and interest-driven learning tasks
Self-directed capacity	Self-regulation; independent learning skills	Enables management of cognitive load and supports sustained learning in complex tasks (Christensen, 2018)	Provide structured scaffolding to support self-directed learning
Learner differences	Confidence; prior knowledge	Shape participation patterns and depth of engagement, influencing learning outcomes	Offer differentiated support to accommodate diverse learner readiness
Gender and inclusion	Gendered confidence; participation tendencies	Socially mediated confidence and participation influence engagement and interaction, potentially leading to unequal learning opportunities (Hoffman, 2012)	Implement gender-responsive strategies to support balanced participation
Instructional engagement	Interactive and practice-oriented learning	Facilitates conceptual understanding by linking theory with practice and reducing abstraction (Sun et al., 2025; Awang-Hashim et al., 2019)	Integrate experiential, multimodal, and applied learning approaches
Assessment and feedback	Feedback quality; assessment clarity	Supports learning regulation, confidence, and strategic adjustment through continuous feedback	Provide timely, clear, and formative feedback

### 3.3 Factors influencing self-directed learning design of flipped classroom teaching

The findings reveal that the effectiveness of self-directed learning (SDL) within flipped classroom (FC) environments is shaped by the interaction between learner readiness, instructional support, and pedagogical structure. These factors collectively determine how students engage with pre-class learning, in-class activities, and knowledge application processes.

Students' learning preferences and individual differences play a central role in shaping their responses to flipped classroom design. The analysis indicates that learners who demonstrate higher levels of autonomy, self-regulation, and confidence are more likely to adapt successfully to SDL-oriented environments, where independent pre-class preparation is required. In contrast, students who are accustomed to teacher-directed instruction often experience difficulty in managing their learning processes, leading to confusion, reduced engagement, and uneven participation (Yilmaz, 2017). Readiness for SDL therefore emerges as a critical condition for effective flipped classroom implementation. This readiness is further influenced by students' ability to organize their learning time, regulate their cognitive efforts, and actively construct knowledge through independent exploration.

Technological familiarity and access also play a significant role in shaping students' engagement with flipped classroom learning. The findings indicate that students' ability to effectively use digital platforms, instructional videos, and online learning tools directly affects their participation in pre-class learning activities. Technological barriers—such as limited digital literacy or unstable access to resources—can reduce motivation and hinder engagement, thereby limiting the effectiveness of SDL-based instructional design (Lai et al., 2021). From an inclusion perspective, disparities in technological access and competence may contribute to unequal learning opportunities, particularly for students with differing levels of prior exposure to digital learning environments.

Instructional design and teacher facilitation play a mediating role in supporting SDL within flipped classroom contexts. The findings show that structured guidance, clear learning pathways, and well-designed instructional materials are essential for enabling students to navigate independent learning tasks. Teachers' roles extend beyond content delivery to include scaffolding, facilitation, and the provision of strategic support that helps students develop self-directed learning competencies. Effective guidance can enhance students' motivation, reduce uncertainty, and support sustained engagement with learning tasks. In addition, collaborative learning opportunities—such as peer discussion and group problem-solving—further support knowledge construction by enabling students to exchange perspectives and co-construct understanding (Algarni and Lortie-Forgues, 2023).

Assessment design and feedback practices further shape students' engagement with flipped classroom learning. The findings indicate that transparent assessment criteria and timely, formative feedback are critical for supporting SDL processes, as they enable students to monitor their progress and adjust their learning strategies. Regular feedback provides learners with direction and reinforces their confidence in managing independent learning tasks. In contrast, unclear assessment expectations may increase uncertainty and reduce engagement. From a gender-responsive perspective, equitable and supportive feedback practices are particularly important, as they can help mitigate differences in confidence and participation by providing consistent guidance across diverse learner groups.

Students' motivation and future-oriented expectations also influence their engagement with flipped classroom learning. The analysis suggests that learners who perceive clear connections between learning tasks and their academic or career goals are more likely to invest effort in SDL activities and actively participate in learning processes (Sergis et al., 2018). Motivation therefore operates not only as an internal driver but also as a factor shaped by instructional design and perceived relevance.

Taken together, these findings demonstrate that effective SDL design in flipped classroom contexts requires alignment between learner readiness, instructional structure, technological support, and assessment practices. These elements operate in an interconnected manner, shaping not only students' engagement with learning tasks but also their confidence, participation, and overall learning experience. Importantly, differences in self-regulation, technological access, and confidence may interact with socially mediated factors, including gendered patterns of participation, to produce uneven learning outcomes. These insights highlight the need for a gender-responsive and inclusion-oriented approach to flipped classroom design, in which instructional strategies are deliberately structured to support diverse learners. Such an approach provides a critical foundation for the development of an integrated framework that combines flipped classroom pedagogy with self-directed learning to promote equitable participation and effective learning in music theory education Table 3.

**Table 3:** Key design mechanisms of sdl in flipped classroom contexts.

Dimension	Core factors	Key mechanisms	Pedagogical implications
Learner readiness	Autonomy; self-regulation; confidence	Determines students' ability to engage in independent pre-class learning and manage learning processes (Yilmaz, 2017)	Provide scaffolding and structured guidance to support SDL readiness
Technological conditions	Digital literacy; access to learning technologies	Enables or constrains participation in pre-class learning and engagement with instructional resources (Lai et al., 2021)	Ensure equitable access and support for technology use
Instructional facilitation	Teacher guidance; scaffolding; collaborative learning	Mediates SDL by supporting knowledge construction, reducing uncertainty, and enhancing engagement (Algarni and Lortie-Forgues, 2023)	Design structured, interactive, and collaborative learning environments
Assessment and feedback	Formative feedback; assessment transparency	Regulates learning processes and supports self-monitoring and strategy adjustment	Provide timely, clear, and formative feedback to guide learning
Motivation and expectations	Learning goals; future-oriented relevance	Influences effort investment and sustained participation in SDL activities (Sergis et al., 2018)	Align learning tasks with meaningful goals and future relevance
Inclusion and gender responsiveness	Confidence differences; participation disparities	Socially mediated factors influence engagement and may lead to unequal learning outcomes	Embed inclusive and gender-responsive design to support equitable participation

### 3.4 Factors influencing acceptance of flipped classroom self-directed learning

The findings indicate that students' acceptance of flipped classroom self-directed learning (FC–SDL) is shaped by the alignment between learner readiness, instructional design quality, and contextual support conditions. Acceptance is not merely a function of exposure to innovative pedagogy but reflects students perceived ability to engage effectively with the demands of self-directed learning and the perceived value of the learning experience.

Learner readiness emerges as a foundational determinant of acceptance. Students who possess stronger self-regulation, time management, and independent learning competencies are more likely to perceive FC–SDL as manageable and beneficial. In contrast, learners with limited experience in autonomous learning may experience uncertainty and reduced confidence when required to engage in pre-class preparation and self-directed knowledge construction. This suggests that acceptance is closely linked to learners perceived self-efficacy and readiness for SDL, rather than the instructional model itself.

Instructional design quality further shapes students' perceptions and acceptance of FC–SDL. The findings show that course designs characterized by clarity, coherence, and engagement—particularly those incorporating interactive and stimulating elements—are more likely to foster positive attitudes toward flipped learning (Birgili et al., 2021). When instructional materials are well-structured and aligned with learning objectives, students are better able to navigate independent learning tasks, which enhances both engagement and acceptance. Conversely, poorly designed or cognitively overwhelming materials may reduce students' willingness to engage with the flipped classroom approach.

Teacher facilitation and social interaction also play a critical role in mediating acceptance. Effective guidance from instructors can reduce uncertainty, support motivation, and enhance students' confidence in managing self-directed learning processes. In addition, collaborative learning environments—characterized by peer discussion, shared problem-solving, and knowledge exchange—contribute to more positive learning experiences and greater acceptance of FC–SDL (Izadpanah, 2022). These findings highlight that acceptance is not solely an individual cognitive process but is socially constructed through interaction and support.

Assessment practices and feedback mechanisms further influence students' acceptance by shaping their perceptions of fairness, transparency, and learning progress. The findings indicate that clearly defined assessment criteria and timely, constructive feedback enable students to better understand expectations and regulate their learning strategies, thereby enhancing their confidence and willingness to engage with FC–SDL (Zheng et al., 2020). In contrast, ambiguous assessment structures may increase anxiety and reduce acceptance.

Contextual conditions, including disciplinary characteristics and learning environments, also affect students' acceptance of flipped classroom approaches. The analysis suggests that the suitability of FC–SDL may vary depending on the complexity and nature of course content, with some learning contexts requiring a balance between independent learning and direct instruction (Goedhart et al., 2019). In addition, supportive learning environments—characterized by adequate resources, institutional support, and positive classroom climates—are associated with higher levels of engagement and acceptance.

From a gender-responsive and inclusion-oriented perspective, acceptance is further influenced by differences in confidence, participation tendencies, and access to learning resources. Students who experience lower confidence or reduced opportunities for participation may be less likely to engage with self-directed learning environments. These differences, shaped by socially mediated experiences, may contribute to uneven acceptance across learner groups if not explicitly addressed. Therefore, inclusive and supportive instructional strategies are essential for ensuring that FC–SDL designs are accessible and acceptable to diverse learners.

Taken together, these findings demonstrate that students' acceptance of FC–SDL is a multidimensional construct shaped by the interaction of learner readiness, instructional design, social support, assessment practices, and contextual conditions. These factors operate in an interconnected manner, influencing not only students' attitudes toward flipped learning but also their willingness to engage in self-directed learning processes. The results underscore the importance of designing FC–SDL environments that align pedagogical structure with learner diversity and provide sufficient support to enhance both acceptance and participation. This provides a critical bridge between instructional design and effective implementation, reinforcing the need for a gender-responsive and inclusive framework for music theory education Table 4.

**Table 4:** Key factors, mechanisms, and pedagogical implications of FC–SDL acceptance.

Dimension	Core factors	Acceptance mechanisms	Pedagogical implications
Learner readiness	Self-regulation; time management; learning autonomy	Higher readiness increases perceived capability and reduces uncertainty in engaging with FC–SDL	Provide preparatory support and scaffolding for SDL readiness
Instructional design quality	Clarity; coherence; engagement of course design	Well-structured and engaging design enhances perceived value and usability of FC–SDL (Birgili et al., 2021)	Design clear, structured, and motivating learning materials
Teacher facilitation & interaction	Guidance; peer collaboration; classroom interaction	Instructional support and social interaction enhance confidence and positive learning experiences (Izadpanah, 2022)	Foster interactive and collaborative learning environments
Assessment and feedback	Transparency; formative feedback	Clear expectations and timely feedback increase confidence and acceptance of learning processes (Zheng et al., 2020)	Provide transparent criteria and continuous feedback
Contextual conditions	Learning environment; disciplinary characteristics; institutional support	Supportive environments and context–pedagogy alignment enhance engagement and acceptance (Goedhart et al., 2019)	Align FC–SDL design with subject characteristics and learning contexts
Inclusion and gender responsiveness	Confidence differences; participation opportunities; access to support	Unequal confidence and participation may reduce acceptance among certain learner groups	Integrate inclusive and gender-responsive strategies to support equitable acceptance

## 4 Discussion

### 4.1 Interpreting the FC–SDL framework

The findings suggest that the effectiveness of flipped classroom self-directed learning (FC–SDL) is best understood as an alignment among learner characteristics, instructional design, and assessment structures, which collectively shape learning processes and outcomes. While prior studies emphasize the flexibility and interactivity of flipped classrooms (Namaziandost and Çakmak, 2020; Paulus Olak Wuwur et al., 2023), the present study demonstrates that pedagogical effectiveness depends on how these conditions support sustained engagement and participation.

Learner characteristics—including motivation, self-regulation, prior knowledge, and confidence—function as foundational inputs that condition engagement in both pre-class and in-class learning. Earlier research often treats these factors as background variables (Gilboy et al., 2015; Blair et al., 2016), whereas the findings here indicate that differences in learner readiness directly shape the depth of engagement and conceptual understanding.

Instructional design and assessment operate as mediating conditions that activate learning processes. Consistent with research on student-centered learning environments (Awidi and Paynter, 2019; Schmid et al., 2023), structured guidance, multimodal resources, and formative feedback support cognitive engagement, strategic learning, and active participation. Learning processes—conceptualized as cognitive engagement, strategic regulation, and participation—serve as mechanisms linking inputs to outcomes, extending prior work by clarifying how engagement translates into deeper understanding and sustained involvement.

Learning outcomes, including conceptual understanding, sustained engagement, and equitable participation, emerge from this interaction. This extends existing research that focuses primarily on performance by incorporating participation and equity as central indicators of learning effectiveness.

### 4.2 Repositioning self-directed learning

The findings reconceptualize self-directed learning (SDL) as a mediated process rather than an individual attribute. While SDL has been widely associated with autonomy and lifelong learning (Boyer et al., 2014), prior studies tend to emphasize learner responsibility. The present study shows that SDL is shaped by instructional guidance, feedback, and interactional support.

This perspective challenges the assumption that learners can independently adapt to flipped environments. Instead, SDL is co-constructed through the interaction between learner agency and pedagogical design, requiring structured scaffolding to support effective engagement (Seherrie, 2023; Mohammadi, 2024).

### 4.3 Integrating gender and inclusion

A key limitation of existing flipped learning research lies in its limited engagement with gender theory, where gender is often treated as a background variable rather than a theoretically grounded construct. In contrast, this study draws on perspectives from gender-responsive pedagogy and social constructivist views of learning, which emphasize that participation, confidence, and interaction are shaped by socially mediated expectations and classroom structures rather than solely by individual traits. From this perspective, gendered patterns of engagement in music theory classrooms can be understood as emerging from interactional norms, feedback practices, and opportunities for participation.

Building on this theoretical grounding, the proposed framework integrates gender not as a demographic category but as a set of instructional and interactional mechanisms embedded within pedagogical design. Inclusive instructional strategies, equitable participation structures, and balanced feedback practices are conceptualized as ways of reshaping learning conditions to support more equitable engagement. This approach aligns with contemporary gender theory by shifting the analytical focus from learner differences to the structuring of learning environments, thereby offering a more robust explanation of how inequities are produced and can be mitigated in music theory education.

### 4.4 Implications for music theory education

The findings highlight the importance of aligning instructional design with the cognitive and motivational demands of music theory learning. Practice-oriented and multimodal approaches can reduce abstraction and support conceptual understanding, while structured feedback enhances learning regulation and confidence.

Attention to inclusion further underscores the need to support diverse learner profiles. Addressing differences in confidence, access, and participation is essential for fostering balanced and effective learning experiences in music theory education.

In music theory education, learning is structured around discipline-specific tasks that require distinct cognitive and procedural processes. For example, harmonic analysis involves identifying chord functions and progressions, counterpoint construction requires the application of rule-based voice-leading principles, and aural training demands the integration of auditory perception and symbolic notation.

Within the FC–SDL model, these tasks are explicitly embedded into instructional design. Pre-class learning supports the acquisition of theoretical rules and analytical procedures, in-class activities emphasize collaborative harmonic analysis and counterpoint problem-solving, while post-class tasks focus on aural dictation and composition-based application. This task-specific structuring ensures that flipped learning directly addresses the unique cognitive and pedagogical demands of music theory.

### 4.5 From framework to practice

**Figure 2:**
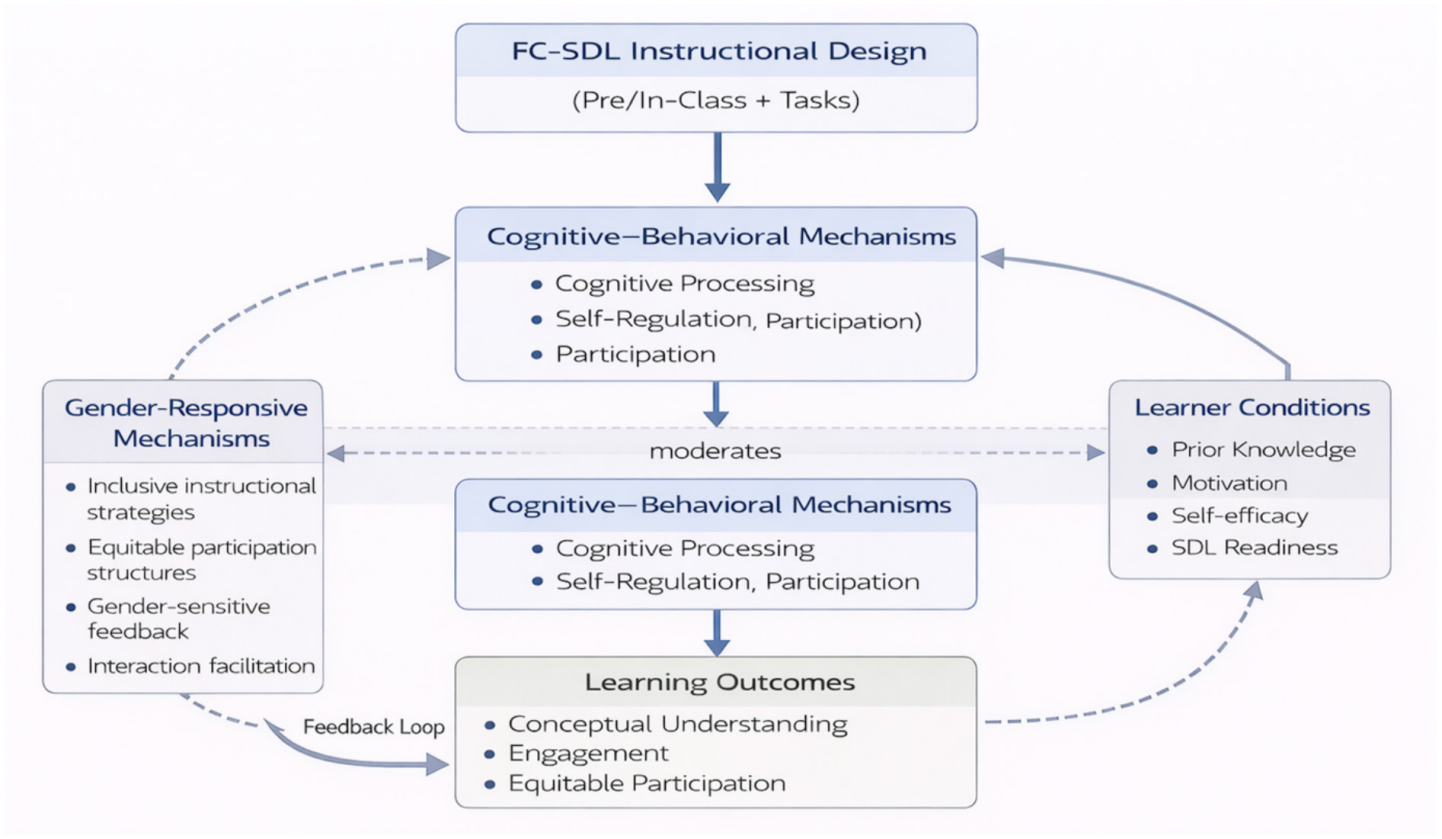
FCF framework for music theory.

The FC–SDL framework presented in Figure 2 establishes a structured and theoretically grounded pathway linking instructional design, cognitive–behavioral mechanisms, and learning outcomes within an inclusion-oriented perspective. Rather than prescribing a fixed instructional model, the framework functions as a flexible yet coherent system in which pedagogical design, learner processes, and contextual factors are dynamically interconnected.

At the core of the framework lies a unidirectional causal sequence. FC–SDL instructional design—comprising pre-class preparation, in-class collaborative activities, and embedded music theory tasks—serves as the primary driver that activates learners' cognitive–behavioral mechanisms. These mechanisms include cognitive processing, self-regulation, and active participation, which collectively mediate the transformation of instructional inputs into meaningful learning experiences.

Importantly, music theory-specific tasks, such as harmonic analysis, counterpoint construction, and aural training, are not treated as peripheral activities but are systematically embedded within the instructional design. By aligning these tasks with both pre-class and in-class phases, the framework ensures that learning activities directly correspond to the cognitive and procedural demands inherent in music theory education, thereby strengthening the explanatory power of the design–mechanism linkage.

Learning outcomes—conceptual understanding, engagement, and equitable participation—are conceptualized as the direct results of these activated mechanisms. In contrast to linear instructional models, the framework incorporates a feedback loop in which learning outcomes recursively inform and refine subsequent instructional design. This feedback structure establishes a closed-loop system that supports continuous pedagogical adaptation and improvement.

Furthermore, the framework explicitly integrates moderating factors that shape the strength and direction of the core relationships. Learner conditions, including prior knowledge, motivation, self-efficacy, and readiness for self-directed learning, influence how effectively instructional design translates into cognitive–behavioral engagement. Simultaneously, gender-responsive mechanisms are conceptualized as instructional and interactional processes—such as inclusive strategies, equitable participation structures, and balanced feedback practices—that shape participation and feedback dynamics within the learning process. This conceptualization is aligned with gender theory perspectives that emphasize the socially constructed and interactionally mediated nature of learning engagement, thereby shifting the focus from gender as a demographic category to gender responsiveness as a pedagogical mechanism.

Taken together, the alignment of instructional design, learning mechanisms, moderating conditions, and feedback processes forms a coherent and logically closed system. This integrated structure provides a theoretically robust foundation for advancing both effectiveness and equity in music theory education, while offering practical guidance for designing inclusive flipped learning environments.

Taken together, the framework provides a coherent integration of instructional design, cognitive–behavioral mechanisms, and gender-responsive processes within a unified and adaptive system. By linking domain-specific tasks with mechanism-driven learning processes and embedding equity considerations within pedagogical interactions, the model moves beyond generic instructional representations and offers a more precise and theoretically integrated account of how effective and inclusive learning in music theory can be achieved.

## 5 Conclusion

This study demonstrates that the effectiveness of flipped classroom self-directed learning (FC–SDL) in music theory education is not an inherent outcome of adopting innovative pedagogy but rather emerges from the dynamic alignment among learner readiness, instructional design, and contextual support conditions. By reconceptualizing self-directed learning as a mediated and co-constructed process, the study extends existing perspectives that position SDL as an individual attribute, instead emphasizing the critical role of structured guidance, interaction, and feedback in shaping meaningful engagement and learning outcomes. Moreover, by integrating gender and inclusion as cross-cutting dimensions, the proposed framework moves beyond conventional effectiveness-oriented approaches and foregrounds equitable participation as a central indicator of pedagogical success, thereby offering a theoretically grounded and integrative model that bridges instructional design, learner diversity, and equity considerations in music education. Nevertheless, several limitations should be acknowledged. The study is based on a conceptual framework development approach grounded in systematic literature synthesis, and thus lacks empirical validation in real classroom contexts, limiting the ability to confirm causal relationships and practical effectiveness. In addition, the scope of the reviewed literature, although comprehensive, is bounded by temporal and database constraints, and the treatment of gender and inclusion, while central, does not fully capture the complexity of intersectional factors such as socio-economic background, cultural context, and prior musical experience. The disciplinary focus on higher education music theory further constrains the generalizability of the findings. Building on these limitations, future research should prioritize empirical validation of the framework through experimental, quasi-experimental, and design-based studies, while also expanding the analytical lens to incorporate intersectional perspectives on learner diversity. Longitudinal investigations are needed to examine how self-directed learning competencies and participation patterns evolve over time, and cross-disciplinary studies may further assess the transferability of the framework to other educational contexts. Additionally, future work should focus on translating the conceptual model into actionable design principles and pedagogical interventions to support the implementation of gender-responsive and inclusive FC–SDL practices. Taken together, this study provides a coherent theoretical foundation and a forward-looking research agenda, contributing to the advancement of both effective and equitable learning in music theory education.
